# Gastrointestinal parasites circulating in wild boars from central Italy and molecular characterization of *Balantioides coli*, *Blastocystis* sp. and *Enterocytozoon bieneusi*

**DOI:** 10.1007/s00436-026-08718-x

**Published:** 2026-07-02

**Authors:** Serena Cavallero, Silvia Rondón, Ilaria Bellini, Isabel Guadano-Procesi, Claudia Chiovoloni, Benedetto Morandi, Stefano Gavaudan, Camilla Sangiovanni, Federica Berrilli, Stefano D’Amelio

**Affiliations:** 1 Department of Public Health and Infectious Diseases, Sapienza University of Rome, 00185 Rome, Italy; 2 Department of Clinical Sciences and Translational Medicine, Tor Vergata University of Rome, 00133 Rome, Italy; 3 Istituto Zooprofilattico Sperimentale dell’Umbria e delle Marche “Togo Rosati”, Perugia, 06126 Italy

**Keywords:** Wild boars, Zoonoses, Gastrointestinal parasites, *Balantioides*, *Blastocystis*, *Enterocytozoon*

## Abstract

The growing abundance and geographic spread of wild boar (*Sus scrofa*) populations in Europe have heightened concerns about their contribution to the transmission of parasites of zoonotic and veterinary importance. This study investigated the occurrence and zoonotic potential of gastrointestinal parasites in 46 wild boars sampled during the 2023 hunting season in Italy. Copromicroscopic analyses revealed that 47.8% of animals were positive for at least one parasite taxon, with frequent coinfections. Thirteen taxa were identified by microscopy, including protozoans (*Entamoeba coli, Iodamoeba butschlii, Giardia duodenalis*) chromists (*Cystoisospora suis, Eimeria* spp., *Balantioides coli, Blastocystis* sp.) and helminths (*Dicrocoelium dendriticum, Ascaris* spp.*, Trichuris* sp.*,* strongyliform larvae, gastrointestinal strongyle-type eggs*, Metastrongylus* spp.), some of zoonotic relevance. Molecular analyses confirmed the presence of *Balantioides coli* and identified *Blastocystis* subtype ST5. Additionally, *Enterocytozoon bieneusi* was molecularly detected in eight animals, representing the first evidence of this microsporidian in wild boars from Italy. Genotypes D, J, and I were identified, with genotype D clustering within the zoonotic Group 1. These findings emphasize the dual epidemiological role of wild boars as reservoirs and sentinel of potential zoonotic parasites and highlight the importance of integrated surveillance strategies within a One Health framework to better assess transmission risks at the wildlife–livestock–human interface.

**Supplementary Information:**

The online version contains supplementary material available at https://doi.org/10.1007/s00436-026-08718-x.

## Introduction

The recent expansion of wild boar (*Sus scrofa*) populations in different parts of the World has raised significant concerns due to its potential negative consequences for biodiversity conservation and economically relevant human activities (Ruiz-Fons 2017). This demographic expansion poses eco-pathological issues and may also represent a threat to both human and animal health. Interactions between humans and wild boars that may favour pathogen transmission to domestic animals may be driven by several factors, such as hunting and activities of game professionals, an increased anthropogenic impact due to the presence of agricultural areas and human settlements in semi-natural areas. Additionally, outdoor activities, socio-economic changes and dietary habits may also be involved. These include the handling of food contaminated with infected stool or the direct manipulation of infected carcasses (Ruiz-Fons 2017).

In Europe, wild boars are implicated in agricultural damages (i.e., crop damages), act as African Swine Fever virus reservoir (Sauter-Louis et al. 2021), and contribute to the transmission of zoonotic pathogens transmission (Altissimi et al. 2024). In 2010, the Italian wild boar population was estimated at around 600,000 animals with a population density of 15 individuals per km^2^ (Massei et al. 2015; Acevedo et al 2022), while unofficial data from the Italian Institute for Environmental Protection and Research, ISPRA, report a population of about 1.5 million. Therefore, considering the relevance of wild boars in the epidemiology of several pathogens of economic relevance and of zoonotic concern, including parasites, surveillance activities and epidemiological screening in these populations are strongly advocated.

In Italy, data on gastrointestinal parasites are still scarce and fragmented; only two recent investigations on parasites circulating in wild boars studied during the regular hunting season or selective culling reported the presence of both protozoans and metazoans (Moretta et al. 2011; Castagna et al. 2019). Among the wide variety of parasites infecting swine, diarrhoeagenic protists are known to cause significant morbidity. In particular, genera such as *Cryptosporidium, Balantioides, Giardia,* and *Blastocystis,* have a well-documented negative impact on swine welfare (Santos Silva et al. 2023). Genetic variants may also imply a zoonotic risk, as reported for *Giardia duodenalis* assemblages A-AI and A-AII (Guadano Procesi et al. 2022), as well as *Blastocystis* subtypes ST15, ST5 and ST3 found in more than 60% of animals analysed (Russini et al. 2020). *Balantioides coli* (syn. *Balantidium coli* and *Neobalantioides coli*) is the only ciliated protist of human and veterinary interest that colonizes the large intestine of several hosts, including humans and pigs, the latter being the dominant reservoir together with wild boars (Santos Silva et al. 2023). As for metazoan infections, high prevalence of *Ascaris suum* and gastrointestinal strongyles, reaching up to 80%, was observed in the southern Italy along with *Trichuris suis*, *Strongyloides ransomi* and *Dicrocoelium dendriticum* (Castagna et al. 2019). The non-zoonotic parasite *Eucoleus garfiai* (syn. *Capillaria garfiai*) has also been recently reported in central and southern Italy (Pacifico et al. 2022; Power et al. 2023).

Microsporidians constitute an additional group of emerging pathogens that colonize porcine intestinal epithelium. According to the recent systematic review by Taghipour et al. (2022), the global prevalence of microsporidian infections in wild boars is estimated to be around 8%. When focusing specifically on *Enterocytozoon bieneusi*, the prevalence appears slightly higher, reaching approximately 10%. About the geographical distribution, the available data show a significant gap concerning Italy.

The study aimed to provide updated data on the occurrence, distribution, and zoonotic potential of pathogens circulating in wild boars in Italy by surveying their gastrointestinal parasites. The study focused on populations inhabiting fragmented habitats in rural areas where the human-wildlife interface is prominent. A copromicroscopic analysis was performed to assess the presence of circulating gastrointestinal parasites and a further molecular approach was carried out on selected taxa of zoonotic interest with the aim of: i) confirming *Balantioides coli* positive samples at the species level; ii) characterizing *Blastocystis* sp. isolates at the subtype and allele levels; iii) detecting and characterizing *E. bieneusi* genotypes in the entire sample set.

## Material and methods

### Copromicroscopy

During 2023, 46 wild boars were sampled in the framework of regular selection hunting and after the official veterinary inspection. In details, animals derived from different hunting grounds located in the central and southern areas of Marche Region (Province of Ancona, Macerata, Fermo and the Monti Sibillini National Park, Supplementary Fig. 1). Immediately after culling, the wild boar carcasses were transported to the game processing center (URCA), and kept in cold storage facilities pending the veterinary inspection. Stool samples were collected from the rectum after evisceration and stored in duplicates for copro-parasitological microscopy (in formalin 10%) and for molecular analysis (in ethanol 70%). Regarding the microscopic analyses, all samples stored in 10% formalin solution were processed with direct smears and flotation. The direct smears with 1% iodine solution and 0.85% saline solution, were performed according to Rondón et al (2023). Two slides were systematically examined by two operators with an optical microscope using 100X, 400X, and 1000X magnifications. Flotation was performed as follows: around 2 g sample was suspended in saline solution and centrifuged at 800 × g for 3 min; after removal of supernatant, the salt–sugar solution (650 ml of warm distilled water, 150 g of salt and 500 g of sugar, SG: 1.28) was added, and another centrifugation at 800xg for 5 min was performed, before adding few drops of the salt-sugar solution until the meniscus formation. After 10 min, a first analysis on the obtained meniscus was carried out for the detection of eggs. Then, around 3 ml, were collected and placed into a new 15 ml Falcon tube for a further flotation step with a second meniscus, to increase the chance to observe cysts and oocysts from protozoans and chromists. After 10 min, the coverslip was placed on a microscope slide with a drop of iodine solution. For each fecal sample processed by flotation, one microscope slide was examined using magnifications at 100X, 400X, and 1000X. Images and measures of cysts, oocysts eggs and larvae were collected with microscope camera lucida for identification, according to the available taxonomic keys (Genchi et al. 2010; World Health Organization 2019). The prevalence of gastrointestinal parasites estimated for all samples was obtained using Quantitative Parasitology software (Rózsa et al. 2000), using 95% of confidence limit according to Clopper Pearson method.

### Molecular analyses

Aliquots stored in 70% ethanol solution were subjected to ethanol removal and a washing step with saline solution before genomic DNA isolation, obtained using the QIAamp Fast DNA Stool Mini Kit (Qiagen), according to the manufacturer’s instructions. Regarding the molecular characterization, samples putatively positive *Balantioides coli* were amplified for the nuclear ribosomal 18S using the primer pairs SSUf- SSUrBB, according to previous studies, describing an expected amplicon of around 1000 bp (Pomajbíková et al. 2013). The assay allows to achieve species identification and discriminate them from *Buxtonella,* a morphologically identical ciliate also potentially present. *Blastocystis* positive samples were amplified with two PCRs for two different regions of the small subunit ribosomal RNA (SSU rRNA or 18S) gene, using the protocols and pair of primers BhRDr-RD5, Blast505-532-Blast998-1017 according to Scicluna et al (2006) and Santín et al (2011), with the aim to define the subtype and the allele.

Moreover, molecular detection for *E. bieneusi* was executed on all samples, and characterization was performed by amplifying the ITS region and parts of the SSU rRNA gene using a nested protocol as previously described (Buckholt et al. 2002), which use one outer primer pair (EBIER1-EBIEF1) and an inner primer pair (EBIER6-EBIEF5) yielding a 390 bp product. For all the PCR reactions, 5 µl of genomic DNA was used in a total reaction volume of 25 µl with MyTaq™ Mix (Meridian, Bioline), and positive and negative controls were included in each reaction. Positive amplicons from *Blastocystis, Balantioides* and *E. bieneusi* were purified using the mi-PCR Purification Kit (Metabion International AG) and sent to an external facility for sequencing (Bio-Fab Research, Rome, Italy). The forward and reverse sequence chromatograms were manually examined and edited using FinchTV software (Geospiza, Inc.; Seattle, WA, USA; http://www.geospiza.com). The consensus sequences were compared with those available in the GenBank database through a Standard Nucleotide BLAST search, to confirm isolate identification. For *Blastocystis*, specimens were identified at subtype and allele level according to the list of STs and alleles maintained at https://pubmlst.org/blastocystis (Jolley et al. 2018). Moreover, a phylogenetic tree was inferred for *E. bieneusi,* to explore the relationships between newly identified and previously reported *E. bieneusi* genotypes. Sequence alignment was performed with AliView (Larsson 2014), using representative reference sequences of the described genotypes (Supplementary Table 1), then a Maximum Likelihood (ML) tree was constructed in MEGA X software (Kumar et al. 2018). The Hasegawa-Kishino-Yano (HKY) (1985) substitution model was selected, according to the lowest AIC and BIC. The reliability of nodes was evaluated through bootstrap analysis with 1000 pseudo-replicates.

## Results

### Copromicroscopy

**Fig. 1:**
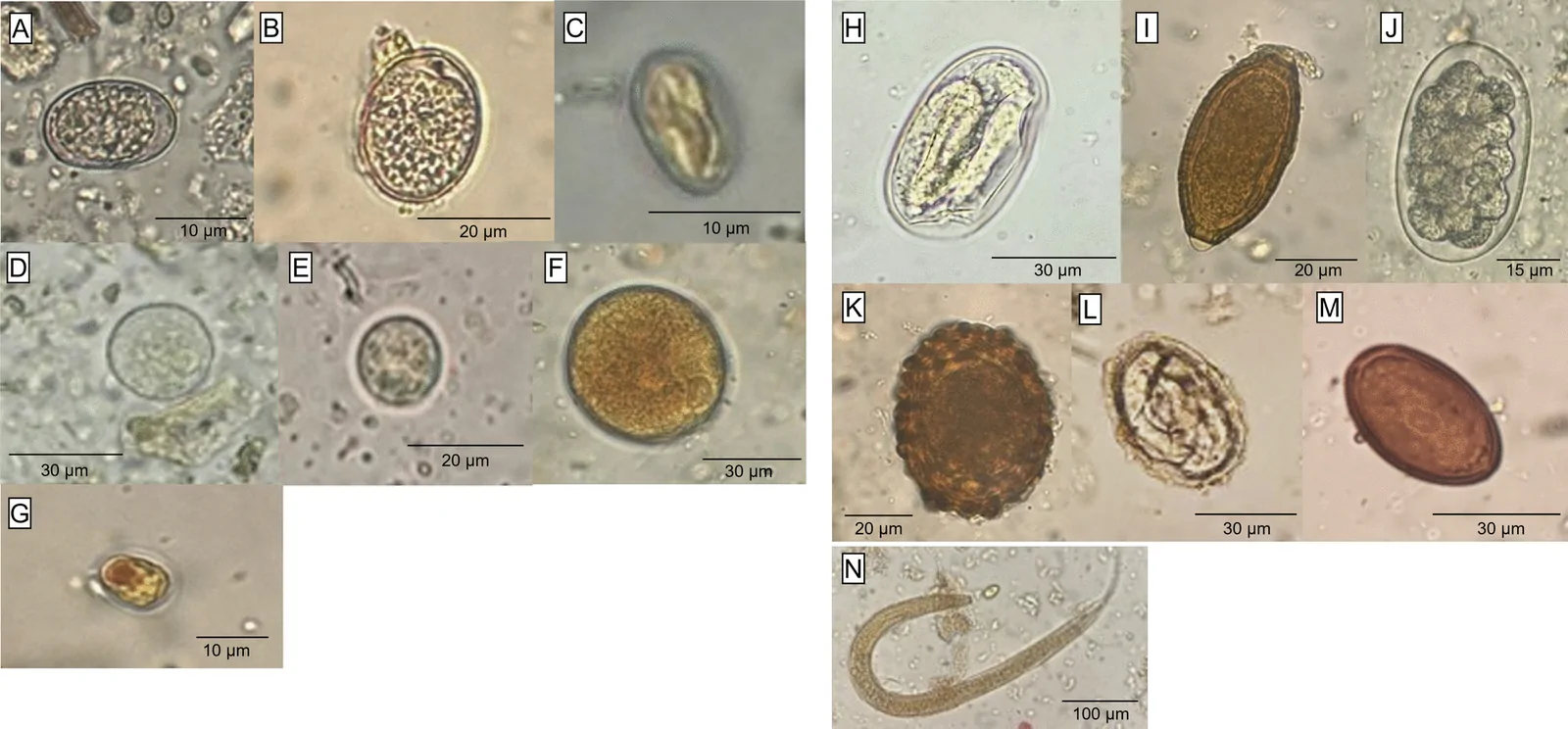
Representative images from the parasite taxa observed by copromicroscopy. **A** Unsporulated *Eimeria* sp. oocyst; **B** Unsporulated *Cystoisospora suis* oocyst; **C** *Giardia duodenalis* cyst; **D** *Entamoeba* sp. cyst; **E** *Blastocystis* sp., **F** *Balantioides coli*; **G** *Iodamoeba butschlii* **H** *Strongyloides ransomi* egg; **I** *Trichuris* sp. **J** Gastrointestinal Strongyle egg; **K** *Ascaris* sp. non-larvated egg; **L** *Metastrongylus* spp. egg; **M** *Dicrocoelium dendriticum* egg; **N** Strongyliform larva

According to copromicroscopic results, approximately half of the wild boars tested positive for gastrointestinal parasites via direct smears and flotation (Table 1). In fact, 22 out of 46 fecal samples analysed were positive at least for 1 parasite taxon (47,8%—CL: 32,9%-63,1%). Samples tested positive for only 1 parasitic taxon were 9 out of 46, and coinfections were also relatively frequent, as 13 animals harboured more than one parasitic taxon (Table 2). Overall, 13 taxonomic entities were found, some of which have zoonotic potential. Taxa included protozoans (*Entamoeba coli, Iodamoeba butschlii, Giardia duodenalis),*chromists (*Cystoisospora suis, Eimeria* spp., *Balantioides coli, Blastocystis* sp.*)* and helminths like trematodes (*Dicrocoelium dendriticum*), nematodes (*Ascaris* sp., *Trichuris* sp., *Strongyloides ransomi,* strongyliform larvae, gastrointestinal strongyle-type eggs, eggs of the lung parasite *Metastrongylus* spp.), (Fig. 1 and Table 1).

**Table 1:** List of protozoans, chromists and metazoans (Class Trematoda and Phylum Nematoda) observed in wild boars through copromicroscopic analysis, with prevalence indicated as percentage along with the number of positive animals and the confidence limit, and the average size in micron of the parasitic forms observed (cysts, oocysts, eggs, larvae)

Protozoans	P % (*n* pos/n tot), CL	Average size (µm)
*Entamoeba coli*	4,3 (2/46), 0,5–14,8	around 25/30
*Iodamoeba butschlii*	2,2 (1/46), 0,1–11,5	10 × 12
*Giardia duodenalis*	2,2 (1/46), 0,1–11,5	11 × 8
Chromists		
*Cystoisospora suis*	17,4 (8/46), 7,8–31,4	20 × 25
*Eimeria* spp.	10,9 (5/46), 3,6–23,6	15 × 12
*Balantioides coli*	15,2 (7/46), 6,3–28,9	55
*Blastocystis* sp.	6,5 (3/46), 1,4–17,9	12 × 14
Trematoda		
*Dicrocoelium dendriticum*	2,2 (1/46), 0,1–11,5	45 × 25
Nematoda		
*Ascaris* spp.	6,5 (3/46), 1,4–17,9	58 × 67
*Trichuris* sp.	6,5 (3/46), 1,4–17,9	57 × 27
Gastrointestinal strongyles	10,9 (5/46), 3,6–23,6	35 × 70
*Strongyloides ransomi*	6,5 (3/46), 1,4–17,9	56 × 33
*Metastrongylus* spp.	2,2 (1/46), 0,1–11,5	56 × 35
Strongyliform larvae	4,3 (2/46), 0,5–14,8	500 × 25

**Table 2:** List of parasites observed in coinfections in wild boar through copromicroscopic analysis, with number of positive animals, number of parasite taxa observed and the parasites

Number of samples	Number of parasite taxa	Coinfections
6	2	*C. suis* and *I. butschlii* *C. suis* and *Trichuris* sp. *C. suis* and *D. dendriticum* *Blastocystis* sp. and *Ascaris* spp. *B. coli* and *Trichuris* sp. *B. coli* and Strongyle egg
5	3	*C. suis, Eimeria* spp. and *S. ransomi* *C. suis, Eimeria* spp. and *Ascaris* spp. *E. coli, Eimeria* spp. and Strongyle egg *B. coli, Eimeria* spp. and Strongyle egg *C. suis, G. duodenalis* and Strongyliform larvae
1	4	*Blastocystis* sp., *Trichuris* sp., *S. ransomi*and *Metastrongylus* sp.
1	5	*E. coli, C. suis, Eimeria* spp.*, B. coli,* and Strongyle egg

### Molecular data

Molecular analyses confirmed the identity of cysts and trophozoites of the ciliate as belonging to the *Balantioides coli* species, in three out of the seven positive animals. Two good consensus sequences were obtained and, by BLAST tool, 100% query coverage and 100% identity was observed with *Balantioides coli* from pigs from Philippines (GQ903678) and from Spain (AM982722), and with *Neobalantidium coli* from primates from several localities. The two sequences were deposited in GenBank under the following accession numbers PX508209-10.

Regarding *Blastocystis* sp. from the three microscopically positive animals*,* all samples provided good amplifications, and two consensus sequences were obtained, confirming the circulation of ST5. One sequence belonged to allele 17 and the other to allele 119. The two sequences were deposited in GenBank under the following accession numbers PX736761-62.

**Fig. 2:**
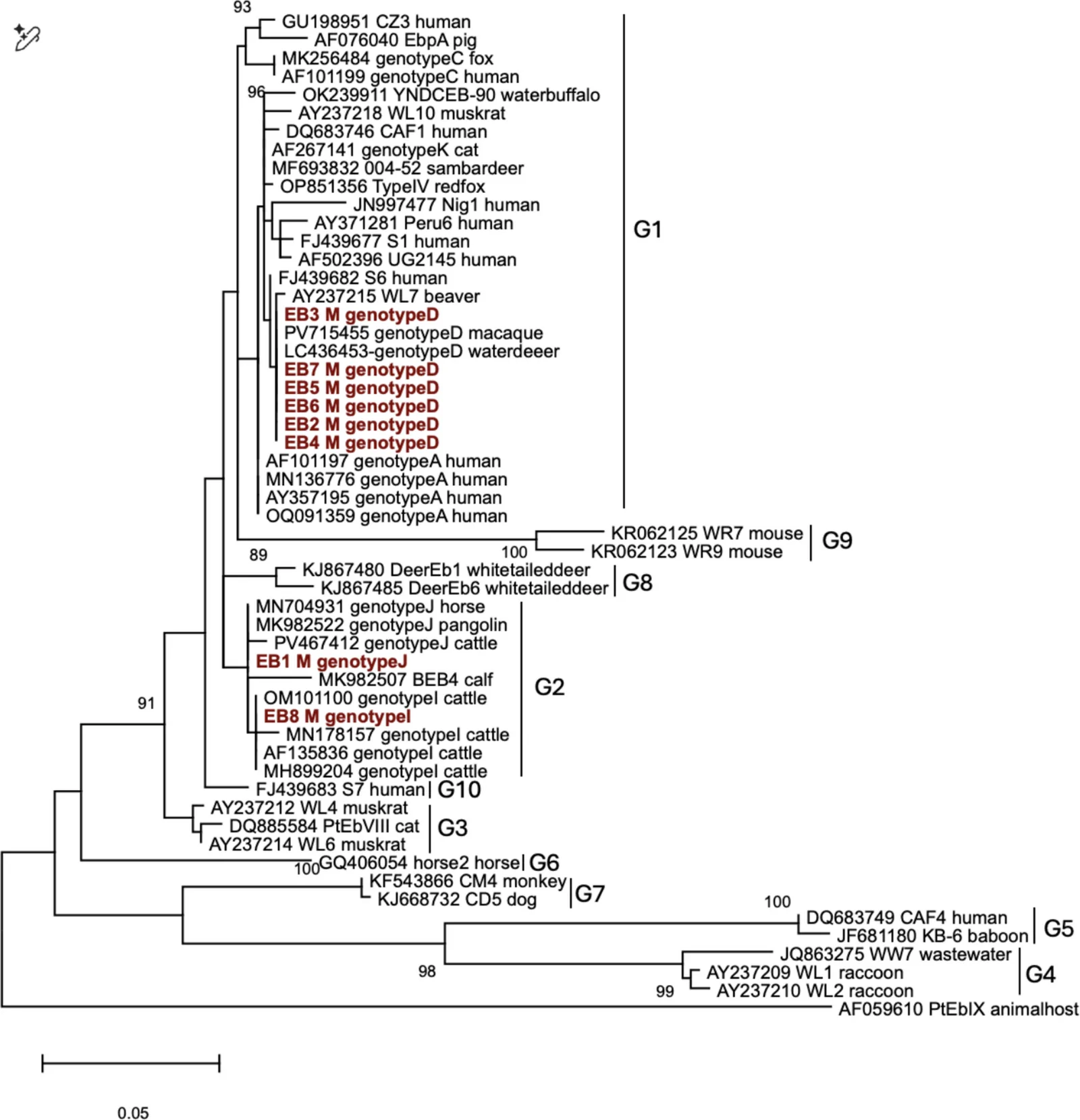
*E. bieneusi* ITS rRNA best consensus phylogenetic tree inferred by the Maximum Likelihood method. Sequences generated in the present study are represented in bold dark red. Genetic distances were computed using the Hasegawa-Kishino-Yano substitution model. Only bootstrap values > 80% are indicated at nodes. Accession numbers of sequences retrieved from GenBank are indicated together with their genotype, host and clustered in groups (see Supplementary Table 1)

The molecular analyses revealed the presence of *E. bieneusi* DNA in the sampled hosts. Specifically, eight specimens tested positive, six were identified as belonging to genotype D, one was classified as genotype J and one as genotype I. Phylogenetic analysis further clarified the evolutionary relationships of these isolates (Fig. 2). The sequences corresponding to genotype D clustered within phylogenetic Group 1, exhibiting high sequence similarity with a sequence previously identified in a water deer (*Hydropotes inermis*) from South Korea. In contrast, the sequences belonging to genotypes J and I were positioned within phylogenetic Group 2, grouping together with several isolates previously reported from a range of host species, including cattle, horses, and a pangolin. The sequences were deposited in GenBank under the following accession numbers PX612230-36 and PX900845.

## Discussion

Passive surveillance of wildlife is the strategy of choice to investigate the health status of wild animals, according to the World Organization for Animal Health (Olivastri et al. 2021; Rossi et al. 2023). In recent years, interest in wild boars has increased considerably as their population growth and adaptation to the urban and peri-urban environments represent a significant risk for both domestic animals and for humans, from a One Health perspective. Despite the limited number of individual hosts and the limited geographical areas here tested, parasites of several taxonomic groups have been identified combining copromicroscopic approach and molecular tools, which allowed to frequently report multiple infections and potentially zoonotic parasites, and to characterize three taxonomic entities, providing an updated and precise information on circulating parasites. However, some sporadic observation may depend on the intrinsic sensitivity issues of conventional microscopy (i.e. intermittent shedding of *Giardia* cysts and morphological polymorphism/fragility of *Blastocystis*).

While intestinal strongyles and eimerian coccidia are so far reported to represent the predominant parasites in wild boars from central Italy (Moretta et al. 2011), in the present study *Balantioides coli* and *Cystoisospora suis* are also reported at high frequency. As for intestinal helminths, the results were consistent with previous data for wild boars from central and southern Italian regions (Moretta et al. 2011; Castagna et al. 2019), although not at a high level of prevalence. Similar trends in the prevalence values for most parasites are also reported from European areas (Băieş et al. 2022; Panayotova-Pencheva and Dakova 2018). In a recent investigation carried out in Poland (Pilarczyk et al. 2024) aimed to compare the prevalence of gastrointestinal parasites circulating in wild boars foraging in urban and suburban areas, a high prevalence of *Ascaris* sp. emerged, particularly in rural areas compared to the city, suggesting serious health threats and economic losses for breeders. Here, although the number of animals infected with *Ascaris* sp. was relatively low, its finding is noteworthy as *Ascaris* infecting swine is traditionally identified as *Ascaris suum.* However, *Ascaris lumbricoides,* the species infecting human*,* as well as the putative hybrid, have been reported to circulate in pigs in Italy (Cavallero et al. 2013; 2021). In Italy, autochthonous human cases of ascariasis are mostly due to *A. suum*, suggesting that zoonotic transmission is predominant over anthroponotic transmission. Therefore, monitoring the occurrence of these parasites should be strongly advised, given the wild boar demographic expansion and their increasing proximity to human settlements.

Overall, the results obtained highlightthe importance of copromicroscopic investigations in obtaining valuable insights into gastrointestinal parasites circulating in wild boars in Italy, reporting taxa belonging to several groups, including protozoans, chromists, and metazoans.

Moreover, the present survey provides novel molecular data on specific parasitic taxa. *Balantioides coli* is widespread and flourishes especially in tropical and subtropical areas where high temperatures and poor sanitary conditions favour its survival and spread. However, it is also frequently reported from temperate areas, thus representing a potential threat to wild and domestic animals, as well as humans worldwide. In fact, contamination and subsequent consumption of untreated water and unsafe handling of pigs’ or other animals’ manure can lead to the acquisition of infections by common parasites in clinically healthy pigs, mainly involving personnel of the food chain (Allievi et al. 2025). Usually, a high prevalence is reported in domestic pigs from free-ranging, small-scale and traditional farms as well as in intensive farms (Santos-Silva et al. 2023). In this study, wild boars are confirmed as an important sylvatic reservoir in which the ciliate was observed in around 15% of tested animals. Given the absence of existing data from Italy, this investigation contributed to fill the gap in knowledge about *B. coli* circulating in wild boars in this country. Such prevalence is consistent with previous reports from Europe (Santos-Silva et al. 2023), in which values reach 45–50% in peculiar conditions (i.e., agricultural land and outdoor pig farms).

The Stramenopiles *Blastocystis* is a ubiquitous chromist found in the intestine of a broad range of human and non-human animal hosts, with unclear clinical significance. Being a highly pleomorphic microorganism with a wide genetic diversity, several subtypes have been described so far with different host affiliations and geographical distributions. Here, ST5 was observed circulating in wild boars from central Italy. ST5 was reported as the most widespread strain in wild boar populations in the Iberian Peninsula, in which all *Blastocystis*-positive animals harboured ST5, also in coinfections with additional STs (Köster et al. 2024). ST5 was reported in wild boars in Italy as well, but with lower frequency compared to ST15, the dominant strain in the studied population, collected from central Italy (Russini et al. 2020). On the other hand, the same study identified ST5 as the most frequent strain in domestic pigs, based on data from a close area compared to the area here investigated. Although ST5 is considered rare in humans, it has been reported to be responsible for a potential zoonosis from pigs in rural China (Yan et al. 2007). Moreover, allele 119 was identified in this study, consistent with previous findings by Russini et al (2020). This observation may provide evidence of a close relationship or of a common source of infection in the area, or suggest a high frequency of a specific variant within a certain strain, which in turn could be related to pathogenicity or host adaptation (Adao and Rivera 2024). The identification of zoonotic STs reinforces the role of wild boars as spreaders of zoonotic infections of public health significance.

Notably, this study represents the first molecular evidence of *E. bieneusi* in wild boars from Italy, thereby updating the geographic distribution of this microsporidian species in Europe. Until now, *E. bieneusi* infections in wild boars were reported exclusively from Central Europe and Spain: in Central Europe, genotype EbpA and other unidentified genotypes were reported (Němejc et al. 2014) and in Spain, Dashti et al. (2020) documented the presence of genotypes EbpA and PigSpEb. In a recent review, among the genotypes identified globally in both domestic pigs and wild boars, the most frequently reported were EbpA, EbpC, and EbpD, with the latter indicated as the most common in domestic pigs worldwide (Taghipour et al. 2022). Another study conducted in 2026 on fattening pigs in Northern Italy highlighted the presence of the genotypes EbpA, KWB4, H, and CC-1/CHPR2 (Allievi et al. 2026).

No prior reports have described the occurrence of genotypes J, I and D in wild boars from any European country. Genotype J has never been identified in either domestic pigs or wild boars worldwide, underscoring the novelty of the findings presented herein. Genotype I was reported in domestic pigs and in cattle in Slovakia (Valenčáková et al. 2019), with several reports also from other European countries, such as Germany, Austria, Czech Republic (recently reviewed in Imre et al. 2025). The consistent detection of genotype D in multiple samples from wild boars in the present study together with the occurrence of genotype I in one specimen could therefore suggest the existence of a potential epidemiological connection between wild and domestic swine populations, mainly present in semi-wild breeding form in the area under study, and often in close contact with cattle farms. These findings may imply the occurrence of cross-species transmission or a shared environmental source of infection, likely facilitated by overlapping habitats, indirect contact, or exposure to contaminated water or feed. While based on a consistent detection pattern, future research incorporating systematic surveillance of livestock and the environment, combined with advanced epidemiological linkage analyses, is crucial to verify this potential connection and fully define infection dynamics.

Phylogeny further corroborated the complex host distribution of *E. bieneusi*. Both genotype D and J have been previously detected in a variety of domestic and wild animals, confirming their broad host range. Importantly, genotype D belongs to Group 1, which encompasses both human and animal isolates, is widely recognized as potentially zoonotic. In contrast, genotypes J and I clustered within Group 2, which predominantly includes sequences derived from animals—especially ruminants—and generally considered non-zoonotic lineages (Guadano Procesi et al. 2025).

## Conclusions

In conclusion, comprehensive copromicroscopical surveillance of parasites in wildlife is essential, particularly in rapidly expanding species such as the wild boar, which can act as a bridge between wild and urban environments, due to its frequent presence at the rural-agricultural-urban interface and its susceptibility to numerous zoonotic and veterinary-relevant diseases. In fact, several potentially zoonotic parasites were reported here, some confirming previous evidence as the circulation of *Ascaris* and *Balantioides*, others expanding the geographic and host range knowledge, as in the case of *E. bieneusi* genotypes in Europe and highlighting the potential epidemiological significance of genotype D as a zoonotic genotype possibly shared between wild boars and domestic pigs. The observation of the genotype I is also of great significance, compelling evidence of a high-risk livestock-wildlife interface, in which the wild boar can be considered as a primary sentinel and *E. bieneusi* report can be interpreted as a proxy species for monitoring the environmental circulation of shared pathogens. This finding emphasizes the need for further surveillance and molecular characterization of *E. bieneusi* in both wildlife and livestock populations.

## Supplementary Information

Below is the link to the electronic supplementary material.

Supplementary file1 (DOCX 2596 KB)

## Data Availability

DNA sequences obtained in the study have been deposited in GenBank.

## References

[CR1] Acevedo P, Aleksovski V, Apollonio M, Berdión O, Blanco-Aguiar J, del Rio L, Ertürk A, Fajdiga L, Escribano F (2022) Wild boar density data generated by camera trapping in nineteen European areas. EFSA Support Publ 19:7214E. 10.2903/sp.efsa.2022.EN-7214

[CR2] Adao DEV, Rivera WL (2024) Subtype-host patterns and genetic differentiation of *Blastocystis* sp. in the Philippines. Heliyon 10:e29019. 10.1016/j.heliyon.2024.e29019 38601700 PMC11004820

[CR3] Akinbo FO, Okaka CE, Omoregie R, Dearen T, Leon ET, Xiao L (2012) Molecular epidemiologic characterization of *Enterocytozoon bieneusi* in HIV-infected persons in Benin City, Nigeria. Am J Trop Med Hyg 86:441–445. 10.4269/ajtmh.2012.11-0548 22403314 PMC3284359

[CR4] Allievi C, Ponce-Gordo F, Villa L, Zanon A, Valleri M, Zanzani SA, Mortarino M, Manfredi MT (2025) Prevalence and molecular characterisation of *Balantioides coli* in pigs raised in Italy. Parasitol Res 124:6. 10.1007/s00436-025-08452-w 39814986 PMC11735580

[CR5] Allievi C, Villa L, Cafiso A, Zanon A, Valleri M, Manfredi MT (2026) First molecular detection and genotyping of *Enterocytozoon bieneusi* (Microsporidia) in fattening pigs from Italy. BMC Vet Res 22:145. 10.1186/s12917-025-05234-5 41546065 PMC12951987

[CR6] Altissimi C, Primavilla S, Roila R, Gavaudan S, Morandi B, Di Lullo S, Coppini M, Baldinelli C, Cai D, Branciari R, Valiani A, Paulsen P, Ranucci D (2024) *Salmonella* in wild boar meat: prevalence and risk assessment in central Italy (Umbria and Marche region). Foods 13:1156. 10.3390/foods13081156 38672829 PMC11049437

[CR7] Amer S, Kim S, Han JI, Na KJ (2019) Prevalence and genotypes of *Enterocytozoon bieneusi* in wildlife in Korea: a public health concern. Parasites Vectors 12:160. 10.1186/s13071-019-3427-6 30961667 PMC6454782

[CR8] Băieş M-H, Boros Z, Gherman CM, Spînu M, Mathe A, Pataky S, Lefkaditis M, Cozma V (2022) Prevalence of swine gastrointestinal parasites in two free-range farms from Nord-West region of Romania. Pathogens 11:954. 10.3390/pathogens11090954 36145387 PMC9506248

[CR9] Breitenmoser AC, Mathis A, Bürgi E, Weber R, Deplazes P (1999) High prevalence of *Enterocytozoon bieneusi* in swine with four genotypes that differ from those identified in humans. Parasitology 118:447–453. 10.1017/s0031182099004229 10363277

[CR10] Breton J, Bart-Delabesse E, Biligui S, Carbone A, Seiller X, Okome-Nkoumou M, Nzamba C, Kombila M, Accoceberry I, Thellier M (2007) New highly divergent rRNA sequence among biodiverse genotypes of *Enterocytozoon bieneusi* strains isolated from humans in Gabon and Cameroon. J Clin Microbiol 45:2580–2589. 10.1128/JCM.02554-06 17537939 PMC1951207

[CR11] Buckholt MA, Lee JH, Tzipori S (2002) Prevalence of *Enterocytozoon bieneusi* in swine: an 18-month survey at a slaughterhouse in Massachusetts. Appl Environ Microbiol 68:2595–2599. 10.1128/AEM.68.5.2595-2599.2002 11976142 PMC127518

[CR12] Castagna F, Musella V, Esposito L, Poerio A, Rinaldi L, Bosco A, Cringoli G, Britti D (2019) Helminths of wild boar (*Sus scrofa*) in the Calabrian Region of Southern Italy. J Wildl Dis 55:416–420. 10.7589/2018-02-028 30311535

[CR13] Cavallero S, Snabel V, Pacella F, Perrone V, D’Amelio S (2013) Phylogeographical studies of *Ascaris* spp. based on ribosomal and mitochondrial DNA sequences. PLoS Negl Trop Dis 7:e2170. 10.1371/journal.pntd.0002170 23593529 PMC3623706

[CR14] Cavallero S, Rondón S, Monterrosa IA, Šnábel V, Papajová I, Goldová M, Štrkolcová G, Caraballo L, Acevedo N, D’Amelio S (2021) Genotyping of *Ascaris* spp. infecting humans and pigs in Italy, Slovakia and Colombia. Infect Genet Evol 94:104997. 10.1016/j.meegid.2021.104997 34252615

[CR15] Chozas M, Dashti A, Prieto-Pérez L, Pérez-Tanoira R, Cobo E, Bailo B, Del Palacio M, Hernández-Castro C, González-Barrio D, Carmena D, Köster PC (2023) *Enterocytozoon bieneusi* and *Encephalitozoon intestinalis* (Microsporidia) in HIV-positive patients in central Spain. Med Mycol 61:myad039. 10.1093/mmy/myad039 37024274

[CR16] Dashti A, Rivero-Juarez A, Santín M, López-López P, Caballero-Gómez J, Frías-Casas M, Köster PC, Bailo B, Calero-Bernal R, Briz V (2020) *Enterocytozoon bieneusi* (Microsporidia): identification of novel genotypes and evidence of transmission between sympatric wild boars (*Sus scrofa ferus*) and Iberian pigs (*Sus scrofa domesticus*) in southern Spain. Trans Emerg Dis 67:2869–2880. 10.1111/tbed.13658 32500974

[CR17] Figueiredo AM, Dashti A, Santín M, Köster PC, Torres RT, Fonseca C, Mysterud A, Carvalho J, Sarmento P, Neves N, Hipólito D, Palmeira JD, Teixeira D, Lima C, Calero-Bernal R, Carmena D (2023) Occurrence and molecular characterization of *Enterocytozoon bieneusi* in wild and domestic animal species in Portugal. Med Mycol 61:myad018. 10.1093/mmy/myad018 36746434

[CR18] Genchi M, Traldi G, Genchi C (2010) Manuale di parassitologia veterinaria. Casa Editrice Ambrosiana, Milano

[CR19] Guadano-Procesi I, Montalbano Di Filippo M, De Liberato C, Lombardo A, Brocherel G, Perrucci S, Di Cave D, Berrilli F (2022) *Giardia duodenalis* in wildlife: exploring genotype diversity in Italy and across Europe. Pathogens 11:105. 10.3390/pathogens11010105 35056053 PMC8777849

[CR20] Guadano-Procesi I, Bosco A, Ciuca L, Pepe P, Sangiovanni C, Di Cave D, Rinaldi L, Berrilli F (2025) *Enterocytozoon bieneusi* in Italian water buffalo calves (*Bubalus bubalis*): An exploratory haplotype analysis within Bovidae family in the European context. Food Waterborne Parasitol 40:e00273. 10.1016/j.fawpar.2025.e00273 40735308 PMC12305309

[CR21] Hasegawa M, Kishino H, Yano T (1985) Dating of the human-ape splitting by a molecular clock of mitochondrial DNA. J Mol Evol 22:160–174. 10.1007/BF02101694 3934395

[CR22] Imre M, Ilie M-S, Florea T, Badea C, Pocinoc A, Imre K (2025) *Enterocytozoon bieneusi* in European domestic ungulates and pets: occurrence, genetic diversity, and public health perspectives from a narrative review. Pathogens 14:1158. 10.3390/pathogens14111158 41305395 PMC12655100

[CR23] Jolley KA, Bray JE, Maiden MCJ (2018) Open-access bacterial population genomics: BIGSdb software, the PubMLST.org website and their applications. Wellcome Open Res 3:124. 10.12688/wellcomeopenres.14826.1 30345391 PMC6192448

[CR24] Karim MR, Wang R, Dong H, Zhang L, Li J, Zhang S, Rume FI, Qi M, Jian F, Sun M, Yang G, Zou F, Ning C, Xiao L (2014a) Genetic polymorphism and zoonotic potential of *Enterocytozoon bieneusi* from nonhuman primates in China. Appl Environ Microbiol 80:1893–1898. 10.1128/AEM.03845-13 24413605 PMC3957649

[CR25] Karim MR, Dong H, Yu F, Jian F, Zhang L, Wang R, Zhang S, Rume FI, Ning C, Xiao L (2014b) Genetic diversity in *Enterocytozoon bieneusi* isolates from dogs and cats in China: host specificity and public health implications. J Clin Microbiol 52:3297–3302. 10.1128/JCM.01352-14 24989604 PMC4313153

[CR26] Karim MR, Rume FI, Li D, Li J, Zhang L (2022) First molecular characterization of *Enterocytozoon bieneusi* in children and calves in Bangladesh. Transbound Emerg Dis 69:1999–2007. 10.1111/tbed.14187 34109760

[CR27] Köster PC, Figueiredo AM, Maloney JG et al (2024) *Blastocystis* occurrence and subtype diversity in European wild boar (*Sus scrofa*) from the Iberian Peninsula. Vet Res 55:133. 10.1186/s13567-024-01385-9 39375799 PMC11460206

[CR28] Kumar S, Stecher G, Li M, Knyaz C, Tamura K (2018) MEGA X: Molecular evolutionary genetics analysis across computing platforms. Mol Biol Evol 35:1547–1549. 10.1093/molbev/msy096 29722887 PMC5967553

[CR29] Larsson A (2014) AliView: a fast and lightweight alignment viewer and editor for large datasets. Bioinformatics 30:3276–3278. 10.1093/bioinformatics/btu531 25095880 PMC4221126

[CR30] Leelayoova S, Subrungruang I, Rangsin R, Chavalitshewinkoon-Petmitr P, Worapong J, Naaglor T, Mungthin M (2005) Transmission of *Enterocytozoon bieneusi* genotype a in a Thai orphanage. Am J Trop Med Hyg 73:104–107. 10.4269/ajtmh.2005.73.104 16014843

[CR31] Li W, Kiulia NM, Mwenda JM, Nyachieo A, Taylor MB, Zhang X, Xiao L (2011) *Cyclospora papionis*, *Cryptosporidium hominis*, and human-pathogenic *Enterocytozoon bieneusi* in captive baboons in Kenya. J Clin Microbiol 49:4326–4329. 10.1128/JCM.05051-11 21956988 PMC3232936

[CR32] Li N, Xiao L, Wang L, Zhao S, Zhao X, Duan L, Guo M, Liu L, Feng Y (2012) Molecular surveillance of *Cryptosporidium* spp., *Giardia duodenalis*, and *Enterocytozoon bieneusi* by genotyping and subtyping parasites in wastewater. PLoS Negl Trop Dis 6:e1809. 10.1371/journal.pntd.0001809 22970334 PMC3435239

[CR33] Li F, Wang R, Guo Y, Li N, Feng Y, Xiao L (2020) Zoonotic potential of *Enterocytozoon bieneusi* and *Giardia duodenalis* in horses and donkeys in northern China. Parasitol Res 119:1101–1108. 10.1007/s00436-020-06612-8 32006227

[CR34] Lobo ML, Xiao L, Cama V, Stevens T, Antunes F, Matos O (2006) Genotypes of *Enterocytozoon bieneusi* in mammals in Portugal. J Eukaryot Microbiol 53(Suppl 1):S61-64. 10.1111/j.1550-7408.2006.00174.x 17169069

[CR35] Massei G, Kindberg J, Licoppe A, Gačić D, Šprem N, Kamler J, Baubet E, Hohmann U, Monaco A, Ozoliņš J et al (2015) Wild boar populations up, numbers of hunters down? A review of trends and implications for Europe. Pest Manag Sci 71:492–500. 10.1002/ps.3965 25512181

[CR36] Mathis A, Bremoser AC, Deplazes P (1999) Detection of new *Enterocytozoon genotypes* in faecal samples of farm dogs and a cat. Parasite 6:189–193. 10.1051/parasite/1999062189 10416194

[CR37] Moretta I, Veronesi F, Di Paola R, Battistacci L, Moretti A (2011) Indagine parassitologica in cinghiali (*Sus scrofa*) cacciati nella stagione venatoria 2009–2010 in Umbria (Italia centrale). Large Anim Rev 17:187–192

[CR38] Němejc K, Sak B, Květoňová D, Hanzal V, Janiszewski P, Forejtek P, Rajský D, Kotková M, Ravaszová P, McEvoy J (2014) Prevalence and diversity of *Encephalitozoon* spp. and *Enterocytozoon bieneusi* in wild boars (*Sus scrofa*) in Central Europe. Parasitol Res 113:761–767. 10.1007/s00436-013-3707-6 24292543

[CR39] Olivastri A, Paoletti B, Lauteri C, Pennisi L, Paludi D, Festino AR, Vergara A (2021) Parasitic cysts in wild boars hunted in Central Italy: the sanitary controls in the wild game meats chain. Ital J Food Saf 10:9383. 10.4081/ijfs.2021.9383 34497779 PMC8404528

[CR40] Onder Z, Yildirim A, Pekmezci D, Duzlu O, Karabulut F, Ozkılıc GN, Pekmezci GZ, Colak ZN, Ciloglu A, Yetismis G, Inci A (2022) Occurrence and molecular characterization of *Enterocytozoon bieneusi* in water buffaloes (*Bubalus bubalis*) in Turkey. Acta Trop 233:106568. 10.1016/j.actatropica.2022.106568 35716763

[CR41] Pacifico L, Sgadari MF, D’Alessio N, Buono F, Restucci B, Sgroi G, Ottaviano M, Antoniciello M, Fioretti A, Tamponi C, Varcasia A, Veneziano V (2022) First description of <i>Eucoleus garfiai</i> (Gallego and Mas-Coma, 1975) in wild boar (<i>Sus scrofa</i>) in Italy. Parasitol Res 121(6):1683–1689. 10.1007/s00436-022-07505-8 35362744 PMC9098567

[CR42] Panayotova-Pencheva M, Dakova V (2018) Studies on the gastrointestinal and lung parasite fauna of wild boars (*Sus scrofa scrofa* L.) from Bulgaria. Ann Parasitol 64:379–384. 10.17420/ap6404.174 30738422

[CR43] Perec-Matysiak A, Buńkowska-Gawlik K, Kváč M, Sak B, Hildebrand J, Leśniańska K (2015) Diversity of *Enterocytozoon bieneusi* genotypes among small rodents in southwestern Poland. Vet Parasitol 214:242–246. 10.1016/j.vetpar.2015.10.018 26520234

[CR44] Perec-Matysiak A, Leśniańska K, Buńkowska-Gawlik K, Merta D, Popiołek M, Hildebrand J (2021) Zoonotic genotypes of *Enterocytozoon bieneusi* in wild living invasive and native carnivores in Poland. Pathogens 10:1478. 10.3390/pathogens10111478 34832633 PMC8619129

[CR45] Pilarczyk B, Tomza-Marciniak A, Pilarczyk R, Felska-Błaszczyk L, Bąkowska M, Udała J, Juszczak-Czasnojć M (2024) A comparison of the prevalence of gastrointestinal parasites in wild boar (*Sus scrofa* L.) foraging in urban and suburban areas. Animals 14:408. 10.3390/ani14030408 38338050 PMC10854758

[CR46] Pomajbíková K, Oborník M, Horák A, Petrželková KJ, Grim JN, Levecke B, Todd A, Mulama M, Kiyang J, Modrý D (2013) Novel insights into the genetic diversity of *Balantidium* and *Balantidium*-like cyst-forming ciliates. PLoS Negl Trop Dis 7(3):e2140. 10.1371/journal.pntd.0002140 23556024 PMC3610628

[CR47] Power K, Martano M, Piscopo N, Viola P, Altamura G, Veneziano V, Carvajal Urueña A, Esposito L (2023) Prevalence of *Eucoleus garfiai* in wild boars hunted at different altitudes in the Campania and Latium regions (Italy). Animals 13:706. 10.3390/ani13040706 36830493 PMC9952325

[CR48] Qi M, Yu F, Zhao A, Zhang Y, Wei Z, Li D, Zhang L (2020) Unusual dominant genotype NIA1 of *Enterocytozoon bieneusi* in children in Southern Xinjiang. China Plos Negl Trop Dis 14:e0008293. 10.1371/journal.pntd.0008293 32569279 PMC7332067

[CR49] Rinder H, Katzwinkel-Wladarsch S, Löscher T (1997) Evidence for the existence of genetically distinct strains of *Enterocytozoon bieneusi*. Parasitol Res 83:670–672. 10.1007/s004360050317 9272556

[CR50] Rinder H, Thomschke A, Dengjel B, Gothe R, Löscher T, Zahler M (2000) Close genotypic relationship between *Enterocytozoon bieneusi* from humans and pigs and first detection in cattle. J Parasitol 86:185–188. 10.1645/0022-3395(2000)086[0185:CGRBEB]2.0.CO;2 10701590

[CR51] Rondón S, Cavallero S, Link A, González C, D’Amelio S (2023) Prevalence and molecular characterisation of *Blastocystis* sp. infecting free-ranging primates in Colombia. Pathogens 12:569. 10.3390/pathogens12040569 37111455 PMC10143058

[CR52] Rossi A, Santi A, Barsi F, Casadei G, Di Donato A, Fontana MC, Galletti G, Garbarino CA, Lombardini A, Musto C, Prosperi A, Pupillo G, Rugna G, Tamba M (2023) Eleven years of health monitoring in wild boars (*Sus scrofa*) in the Emilia-Romagna Region (Italy). Animals 13(11):1832. 10.3390/ani13111832 37889705 PMC10252029

[CR53] Rózsa L, Reiczigel J, Majoros G (2000) Quantifying parasites in samples of hosts. J Parasitol 86:228–232. 10.1645/0022-3395(2000)086[0228:QPISOH]2.0.CO;2 10780537

[CR54] Ruiz-Fons F (2017) A review of the current status of relevant zoonotic pathogens in wild swine (*Sus scrofa*) populations: changes modulating the risk of transmission to humans. Transbound Emerg Dis 64:68–88. 10.1111/tbed.12369 25953392

[CR55] Russini V, Di Filippo MM, Fanelli R, Polidori M, Berrilli F, Di Cave D, Novelletto A, Calderini P (2020) Characterization of prevalence and genetic subtypes of *Blastocystis* sp. in wild and domestic Suidae of central Italy aided by amplicon NGS. Vet Parasitol Reg Stud Rep 22:100472. 10.1016/j.vprsr.2020.100472 33308752

[CR56] Sak B, Brady D, Pelikánová M, Květoňová D, Rost M, Kostka M, Tolarová V, Hůzová Z, Kváč M (2011) Unapparent microsporidial infection among immunocompetent humans in the Czech Republic. J Clin Microbiol 49:1064–1070. 10.1128/JCM.01147-10 21191056 PMC3067711

[CR57] Santín M, Vecino JA, Fayer R (2010) A zoonotic genotype of *Enterocytozoon bieneusi* in horses. J Parasitol 96:157–161. 10.1645/GE-2184.1 19799490

[CR58] Santín M, Gómez-Muñoz MT, Solano-Aguilar G, Fayer R (2011) Development of a new PCR protocol to detect and subtype *Blastocystis* spp. from humans and animals. Parasitol Res 109:205–212. 10.1007/s00436-010-2244-9 21210149

[CR59] Santin M, Fayer R (2015) *Enterocytozoon bieneusi*, *Giardia*, and *Cryptosporidium* infecting white-tailed deer. J Eukaryot Microbiol 62:34–43. 10.1111/jeu.12155 25066778

[CR60] Santos-Silva S, Moraes DFDSD, López-López P, Palmeira JD, Torres RT, São José Nascimento M, Dashti A, Carmena D, Rivero-Juarez A, Mesquita JR (2023) Survey of zoonotic diarrheagenic protist and Hepatitis E Virus in wild boar (*Sus scrofa*) of Portugal. Animals (Basel) 13:256. 10.3390/ani13020256 36670797 PMC9854796

[CR61] Sauter-Louis C, Conraths FJ, Probst C, Blohm U, Schulz K, Sehl J, Fischer M, Forth JH, Zani L, Depner K, Mettenleiter TC, Beer M, Blome S (2021) African swine fever in wild boar in Europe: A review. Viruses 13:1717. 10.3390/v13091717 34578300 PMC8472013

[CR62] Scicluna SM, Tawari B, Clark CG (2006) DNA barcoding of *Blastocystis*. Protist 157:77–85. 10.1016/j.protis.2005.12.001 16431158

[CR63] Sulaiman IM, Bern C, Gilman R, Cama V, Kawai V, Vargas D, Ticona E, Vivar A, Xiao L (2003a) A molecular biologic study of *Enterocytozoon bieneusi* in HIV-infected patients in Lima, Peru. J Eukaryot Microbiol 50:591–596. 10.1111/j.1550-7408.2003.tb00642.x 14736175

[CR64] Sulaiman IM, Fayer R, Lal AA, Trout JM, Schaefer FW 3rd, Xiao L (2003b) Molecular characterization of microsporidia indicates that wild mammals Harbor host-adapted *Enterocytozoon* spp. as well as human-pathogenic *Enterocytozoon bieneusi*. Appl Environ Microbiol 69:4495–4501. 10.1128/AEM.69.8.4495-4501.2003 12902234 PMC169096

[CR65] Taghipour A, Bahadory S, Khazaei S, Zaki L, Ghaderinezhad S, Sherafati J, Abdoli A (2022) Global molecular epidemiology of microsporidia in pigs and wild boars with emphasis on *Enterocytozoon bieneusi*: a systematic review and meta-analysis. Vet Med Sci 8:1126–1136. 10.1002/vms3.751 35113502 PMC9122395

[CR66] ten Hove RJ, Van Lieshout L, Beadsworth MB, Perez MA, Spee K, Claas EC, Verweij JJ (2009) Characterization of genotypes of *Enterocytozoon bieneusi* in immunosuppressed and immunocompetent patient groups. J Eukaryot Microbiol 56:388–393. 10.1111/j.1550-7408.2009.00393.x 19602086

[CR67] Tumwine JK, Kekitiinwa A, Nabukeera N, Akiyoshi DE, Buckholt MA, Tzipori S (2002) *Enterocytozoon bieneusi* among children with diarrhea attending Mulago Hospital in Uganda. Am J Trop Med Hyg 67:299–303. 10.4269/ajtmh.2002.67.299 12408671

[CR68] Valenčáková A, Danišová O (2019) Molecular characterization of new genotypes *Enterocytozoon bieneusi* in Slovakia. Acta Trop 191:217–220. 10.1016/j.actatropica.2018.12.031 30586572

[CR69] World Health Organization (2019) Bench aids for the diagnosis of intestinal parasites, 2nd edn. WHO, Geneva

[CR70] Yan Y, Su S, Ye J, Lai X, Lai R, Liao H, Chen G, Zhang R, Hou Z, Luo X (2007) *Blastocystis* sp. subtype 5: a possibly zoonotic genotype. Parasitol Res 101:1527–1532. 10.1007/s00436-007-0672-y 17665214

[CR71] Zhang Y, Koehler AV, Wang T, Haydon SR, Gasser RB (2018) First detection and genetic characterisation of *Enterocytozoon bieneusi* in wild deer in Melbourne’s water catchments in Australia. Parasit Vectors 11:2. 10.1186/s13071-017-2577-7 29295716 PMC5751821

[CR72] Zhang Y, Koehler AV, Wang T, Haydon SR, Gasser RB (2019) *Enterocytozoon bieneusi* genotypes in cattle on farms located within a water catchment area. J Eukaryot Microbiol 66:553–559. 10.1111/jeu.12696 30358006

