# Supplementary material for: Gastrointestinal parasites circulating in wild boars from central Italy and molecular characterization of Balantioides coli, Blastocystis sp. and Enterocytozoon bieneusi
Source: Parasitol Res. 2026 Jul 2;125(1):112. doi: 10.1007/s00436-026-08718-x (PMC13597627; doi:10.1007/s00436-026-08718-x)
Supplement: Supplementary file 1 [file 436_2026_8718_MOESM1_ESM.docx]

Supplementary Information – Parasitology Research

**Gastrointestinal parasites circulating in wild boars from central Italy and molecular characterization of *Balantioides coli*, *Blastocystis sp.* and *Enterocytozoon bieneusi***

Serena Cavallero^1^, Silvia Rondón^1^, Ilaria Bellini^1^, Isabel Guadano Procesi^2^, Claudia Chiovoloni^1^, Benedetto Morandi^3^, Stefano Gavaudan^3^, Camilla Sangiovanni^2^, Federica Berrilli^2^, Stefano D’Amelio^1^

1.Department of Public Health and Infectious Diseases, Sapienza University of Rome, 00185 Rome, Italy

2.Department of Clinical Sciences and Translational Medicine, Tor Vergata University of Rome, 00133 Rome, Italy

3. Istituto Zooprofilattico Sperimentale dell'Umbria e delle Marche ''Togo Rosati'', 06126, Perugia, Italy.

Corresponding Stefano D’Amelio [stefano.damelio@uniroma1.it](mailto:stefano.damelio@uniroma1.it) ORCID: 0000-0002-1871-9553

**Supplementary Figure1:** Study area **-** animals derived from different hunting grounds located in the central and southern areas of Marche Region (Province of Ancona, Macerata, Fermo and the Monti Sibillini National Park). The map was created using the Geographic Information System software QGIS 3.22.0.

**Supplementary Table1:** list of reference sequences of *Enterocytozoon bieneusi* genotypes retrieved in GenBank and used to infer phylogenetic tree. In details: Accession numbers, isolate name, genotype, host species and literature references are provided.

| **Accession Number** | **Isolate** | **Genotype** | **Host** | **Reference** |
| --- | --- | --- | --- | --- |
| LC436453 | GyungNam_WD03 | D | Water deer | Amer *et al*., 2019 |
| PV715455 | H22 | D | Macaque | - |
| AY237215 | 3599 | WL7 | Beaver | Sulaiman *et al*., 2003b |
| JN997477 | 25121-Nig1 | Nig1 | Human | Akinbo *et al.,* 2012 |
| FJ439682 | - | S6 | Human | ten Hove *et al*., 2009 |
| OK239911 | WbEb3 | YNDCEB-90 | Water buffalo | Onder *et al*., 2022 |
| DQ683746 | G0084 | CAF1 | Human | Breton *et al*., 2007 |
| AY237218 | 5489 | WL10 | Muskrat | Sulaiman *et al*., 2003b |
| OP851356 | RMAL5 | Type IV | Red fox | Figueiredo *et al*., 2023 |
| AF267141 | - | K | Cat | - |
| MF693832 | OS1917 | 004-52 | Sambar deer | Zhang *et al.* 2018 |
| AF101197 | - | A | Human | Rinder *et al*., 1997 |
| MN136776 | 561 | A | Human | Qi *et al*., 2020 |
| AY357195 | - | A | Human | Leelayoova *et al*., 2005 |
| OQ091359 | 11VIH | A | Human | Chozas *et al.*, 2023 |
| FJ439677 | - | S1 | Human | ten Hove *et al*., 2009 |
| AF502396 | UG2145 | UG2145 | Human | Tumwine *et al*., 2002 |
| AY371281 | 7939 | Peru6 | Human | Sulaiman *et al*., 2003a |
| AF076040 | - | EbpA | Pig | Breitenmoser *et al*., 1999 |
| GU198951 | 2288 | CZ3 | Human | Sak *et al*., 2011 |
| AF101199 | - | C | Human | Rinder *et al*., 1997 |
| MK256484 | L10 | C | Red fox | Perec-Matysiak *et al*., 2021 |
| KR062123 | 8-S-11F | WR9 | Mouse | Perec-Matysiak *et al*., 2015 |
| KR062125 | 28-M-11S | WR7 | Mouse | Perec-Matysiak *et al*., 2015 |
| MN178157 | HN5 | I | Cattle | - |
| OM101100 | JS96 | I | Cattle | - |
| MH899204 | FarmB_51 | I | Cattle | Zhang *et al.* 2019 |
| AF135836 | - | I | Cattle | Rinder *et al*., 2000 |
| MK982522 | ZP-192 | J | Pangolin | - |
| MN704931 | SCAU8912J | J | Horse | Li *et al*., 2020 |
| PV467412 | LC EB-11 | J | Cattle | - |
| MK982507 | PC53 | BEB4 | Calf | Karim *et al*., 2022 |
| KJ867480 | 4 | DeerEb1 | Deer | Santín *et al*., 2015 |
| KJ867485 | 52 | DeerEb6 | Deer | Santín *et al*., 2015 |
| AY237212 | 3666 | WL4 | Muskrat | Sulaiman *et al*., 2003b |
| AY237214 | 5540 | WL6 | Muskrat | Sulaiman *et al*., 2003b |
| DQ885584 | PtEb VIII | PtEb VIII | Cat | Lobo *et al*., 2006 |
| FJ439683 | - | S7 | Human | ten Hove *et al*., 2009 |
| GQ406054 | - | Horse2 | Horse | Santín *et al*., 2010 |
| KF543866 | S-147 | CM4 | Gollden snub-nosed monkey | Karim *et al*., 2014a |
| KJ668732 | ZD-380 | CD5 | Dog | Karim *et al*., 2014b |
| DQ683749 | G0475 | CAF4 | Human | Breton *et al*., 2007 |
| JF681180 | 27920 | KB-6 | Baboon | Li *et al*., 2011 |
| JQ863275 | 19107 | WW7 | Waste water | Li *et al*., 2012 |
| AY237209 | 5496 | WL1 | Raccoon | Sulaiman *et al*., 2003b |
| AY237210 | 3608 | WL2 | Raccoon | Sulaiman *et al*., 2003b |
| AF059610 | - | PtEBIX | Animal host | Mathis *et al*., 1999 |
